# Letter from W. B. Witt, 1st Assistant and Acting-Surgeon, 69th Ind. Vol. Infantry

**Published:** 1863

**Authors:** W. B. Witt

**Affiliations:** 1st Assistant and Acting-Surgeon; Ion Plantation, La., Head-Quarters 69th Indiana Vol. Infantry


ION PLANTATION, LA.,
HEAD-QUARTERS 69TH INDIANA VOL. INFANTRY,
*April 13th*, 1863.

EDITOR OF MEDICAL EXAMINER,

DEAR SIR:—The following case may be of interest to you and your readers:—

Mr. S., Co. G., a teamster, on the 10th of April, whilst removing a wagon-hammer, received a blow from it upon the left super ciliary ridge, inflicting an incised contused wound about one inch in length. He was knocked down by the blow, but, by the time I reached him, was sitting up, and complained a little of his head aching; but, as it was nearly night, thought that by sleeping he would be all right in the morning. In the morning, he went to work as usual, and made no farther complaint until the evening of the 13th, when he came to me and desired me to dress the wound with adhesive-plaster, as he found the water dressing rather inconvenient. In conversing with him, I found that he had a slight headache most of the time, but nothing serious, and, further, that he could not see so as to distinguish one object from another; but there was nothing in the appearance of the eye to indicate anything remarkable, except that it was considerably ecchymosed.

On the evening of the 14th, whilst carrying a bucket of water, he fell and expired after drawing only one or two breaths.

*Autopsy.*—April 15th, fifteen hours after death:—The posterior part of the scalp was well-filled with blood, but the tissues were not contused. On opening the cranium, the cerebellum seemed in a healthy condition, with no inflammatory indications at any point. On opening the ventricles, a small clot, about the size of a crow's quill, was found in each ventricle. On raising the tentorium cerebelli, I found the cavity beneath very full of liquid blood; and, on removing the cerebellum, a small fissure was found in the right bassilar vein, about half a-line in length; and also the internal and posterior portion of petrous part of the temporal bone fractured about one inch in length. In haste, I am yours truly, W. B. WITT,

1*st Assistant and Acting-Surgeon*, 69*th Ind. Vol. Infantry.*

